# Monoolein-Based Wireless Capacitive Sensor for Probing Skin Hydration

**DOI:** 10.3390/s24144449

**Published:** 2024-07-10

**Authors:** Vivek Chaturvedi, Magnus Falk, Sebastian Björklund, Juan F. Gonzalez-Martinez, Sergey Shleev

**Affiliations:** 1 Department of Biomedical Science, Faculty of Health and Society, Malmö University, 20506 Malmö, Sweden; vivek.chaturvedi@mau.se (V.C.); sebastian.bjorklund@mau.se (S.B.); juan.fransisco.gonzales@mau.se (J.F.G.-M.); 2 Biofilms Research Center for Biointerfaces, Malmö University, 20506 Malmö, Sweden; 3 Department of Applied Physics and Naval Technology, Polytechnical University of Cartagena, 30202 Cartagena, Spain

**Keywords:** humidity sensor, wireless device, amphiphilic film, monoolein, skin hydration

## Abstract

Capacitive humidity sensors typically consist of interdigitated electrodes coated with a dielectric layer sensitive to varying relative humidity levels. Previous studies have investigated different polymeric materials that exhibit changes in conductivity in response to water vapor to design capacitive humidity sensors. However, lipid films like monoolein have not yet been integrated with humidity sensors, nor has the potential use of capacitive sensors for skin hydration measurements been fully explored. This study explores the application of monoolein-coated wireless capacitive sensors for assessing relative humidity and skin hydration, utilizing the sensitive dielectric properties of the monoolein–water system. This sensitivity hinges on the water absorption and release from the surrounding environment. Tested across various humidity levels and temperatures, these novel double functional sensors feature interdigitated electrodes covered with monoolein and show promising potential for wireless detection of skin hydration. The water uptake and rheological behavior of monoolein in response to humidity were evaluated using a quartz crystal microbalance with dissipation monitoring. The findings from these experiments suggest that the capacitance of the system is primarily influenced by the amount of water in the monoolein system, with the lyotropic or physical state of monoolein playing a secondary role. A proof-of-principle demonstration compared the sensor’s performance under varying conditions to that of other commercially available skin hydration meters, affirming its effectiveness, reliability, and commercial viability.

## 1. Introduction

Wireless humidity sensors, which utilize Radio Frequency, Bluetooth, or Wi-Fi technologies, represent a significant advancement in control systems [1,2,3,4,5,6,7,8] across various applications. These devices wirelessly measure relative humidity (RH) levels, offering easier installation and greater flexibility compared to traditional wired sensors. They are crucial in industries such as microfabrication, agriculture, pharmaceuticals, and food production, where humidity significantly influences quality. These sensors facilitate continuous condition monitoring and, when integrated into smart systems, enable automatic responses to changes in humidity. This integration increases operational efficiency and reduces the need for manual intervention. Recent advancements in materials and microfabrication techniques have led to the development of smaller, more energy-efficient, and precise sensors [9,10].

Wireless sensor networks (WSNs) are comprised of sensor nodes that sense, collect, and gather data continuously, providing a convenient platform for different applications, e.g., healthcare monitoring [11,12,13,14,15]. By incorporating Internet of Things principles, these sensors become part of a larger network of devices, enabling comprehensive monitoring and analysis [16,17,18,19,20,21]. As wireless technology continues to evolve, these sensors are acquiring sophisticated features, such as long-range transmission and integration with cloud-based data analytics, expanding their potential applications and driving innovation in the field [22,23,24,25,26,27].

Skin sensing is a burgeoning technology, predominantly utilized in specialized applications and advanced wearable devices [28,29,30,31,32,33]. Miniature wireless skin sensors are engineered to measure and monitor skin hydration (moisture levels) and find applications in various sectors, including healthcare, sports, and cosmetics. In medical environments, these sensors are instrumental for monitoring skin conditions, aiding in wound healing, and managing skin diseases such as eczema [34,35,36,37]. Athletes and fitness enthusiasts may use these sensors to track sweat levels and hydration status during physical activities [38,39,40,41,42,43]. Additionally, in the cosmetic industry, these sensors facilitate personalized skincare by assessing the skin’s hydration level to recommend suitable products [30,44,45]. Skin hydration sensors could be integrated into various wearable devices such as smartwatches, fitness bands, or smart clothing [46,47]. Ongoing research and development in wearable electronics have resulted in more sophisticated, compact, and user-friendly skin sensors [48,49,50,51]. Although these sensors are becoming increasingly common, they may still be priced at the higher end of the spectrum or embedded in more expensive devices [52,53]. Availability might also vary based on region and specific use cases [54,55]. Typically, these devices connect wirelessly to smartphones or computers, where specialized applications or software analyze the data to provide insights. However, a robust sensing platform that demonstrates repeatability and low hysteresis, ensuring reliable data collection over time and enhanced by remote sensing capabilities, is still required.

A significant portion of humidity sensors designed for specific applications are capacitive humidity sensors (CHSs) [56]. Typically, CHSs consist of interdigitated electrodes (IDEs) coated with a dielectric layer sensitive to varying RH levels. Previous studies have explored ceramic-based and polymer-based CHSs [57]. Polymeric materials exhibit changes in conductivity in response to water vapor. For instance, water vapor diffuses into polymeric matrices such as polyimide, poly (methyl methacrylate), and cellulose acetate butyrate films deposited on IDEs, altering dielectric constant (*ε*), which can be detected as a change in capacitance [8]. However, lipid films like monoolein (MO) have not yet been integrated with CHSs, nor has the potential use of CHSs for skin hydration (SH) measurements been fully explored.

**Figure 1:**
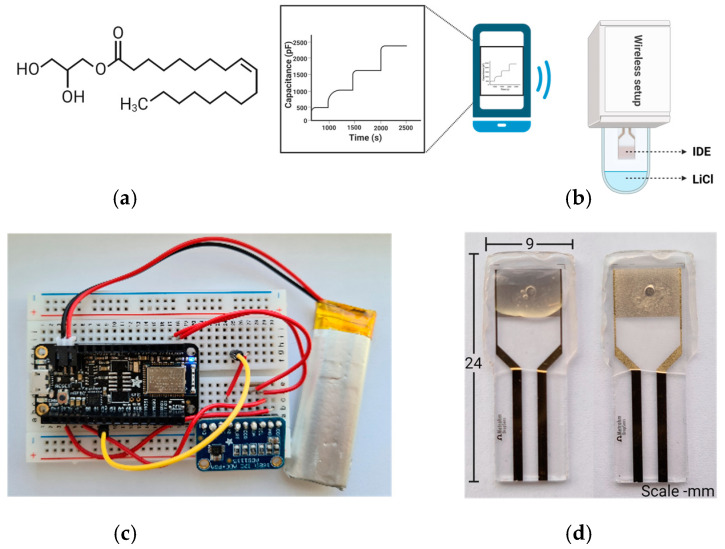
(**a**) Chemical structure of monoolein. (**b**) Schematic illustration showing wireless Adafruit nRF52832 BLE setup for continuous capacitance measurements, where change of capacitance resulting from different molal concentrations of LiCl was continually measured (created with BioRender.com). (**c**) Photograph of the wireless Adafruit nRF52832 microcontroller coupled with ADS 1115 digital-to-analog converter. (**d**) Photographs of laminated IDE dry (right) and covered with MO droplets (left).

In the current work, we report a wireless humidity sensor featuring a lipid film as the active component, which is sensitive to changes in RH across selected ranges at various temperatures. MO has been utilized in various applications due to its inherent physicochemical properties (Figure 1a) [58,59]. The lipid film undergoes hydration-induced changes, followed by the sorption and desorption of water molecules at varying RH values [60]. We observed a relationship between capacitance changes and different RH levels when IDEs coated with MO were exposed to varying humidity. To demonstrate the practical application of our sensor, we attached it to the ring finger of volunteers’ left hands and conducted capacitive measurements under a range of skin temperature (ST) and skin hydration (SH) levels. For comparison and control, we used two commercially available devices: the wireless BG20 from Yones Toptech, which can measure both skin and ambient temperature and humidity, and the wire-based Dermalab^®^ Combo hydration probe from Cortex Technology, which measures SH. Our experimental design facilitated a direct comparison to evaluate the performance and accuracy of the developed sensor under real-world conditions.

## 2. Materials and Methods

### 2.1. Chemicals

Monoolein (MO, 98% grade, CAS no. 111-03-5) was purchased from Danisco (Copenhagen, Denmark). Anhydrous LiCl (99% grade, CAS no. 7447-41-8) and ethanol (99%) were obtained from Sigma-Aldrich (St. Louis, MO, USA). All LiCl solutions were prepared with ultrahigh-quality water (UHQ; resistivity, 18.2 MΩ·cm) obtained using an Elgastat UHQ II device from Elga Ltd. (High Wycombe, Bucks, UK). Dialysis membranes with average width 10 mm were purchased from Sigma-Aldrich (St. Louis, MO, USA).

### 2.2. Quartz Crystal Microbalance with Dissipation (QCM-D)

Water sorption isotherms and dissipation data from thin films of MO were obtained using a quartz crystal microbalance with dissipation monitoring (QCM-D), equipped with a humidity module, following the methodology previously described [61]. The QCM-D technique works by inducing resonance in a piezoelectric quartz sensor through the application of an alternating voltage and monitoring the frequency of the resulting oscillating shear motion. Any change in mass adsorbed onto the sensor leads to a change in frequency, enabling the accurate determination of the adsorbed mass on the sensor surface via the Sauerbrey equation, Equation (1) [62]. Equation (1) assumes that the adsorbed mass is significantly smaller than the mass of the crystal, that the material is rigidly adsorbed, and that it is uniformly distributed across the active area of the crystal. Additionally, the viscoelastic properties of the adsorbed material are analyzed through changes in the dissipation data, which reflect alterations in the decay time of the oscillating resonator when the alternating potential is deactivated.

(1) $$-\frac{\Delta f}{n}=\frac{2f_{0}^{2}m_{f}}{Z_{q}}$$

A Q-sense E4 instrument, equipped with the humidity module QHM 401 and AT-cut SiO2 (QSX 303, 5 MHz) sensors, was used (Biolin Scientific AB, Gothenburg, Sweden). Before coating the sensor with MO, the sensor was washed with UHQ water and sodium dodecyl sulfate (2 wt%) as described in the *q*-sense guideline manual (cleaning protocols B and A-I for QSX 303 and 301, respectively). Baselines of uncoated and cleaned sensors were measured in a dry N_2_ atmosphere at 25 °C. Next, the sensor was spin coated with 10 μL of MO solution 2% (*w*/*v*) dissolved in ethanol, and the coated sensor was vacuum dried overnight. Next, the sensor was mounted in the humidity module and further dried by flowing N_2_ gas until a stable baseline of the frequency was obtained. Next, the water sorption experiment was initiated by flowing LiCl solutions with defined water activity (*a*_w_) values through the humidity module. Since only water vapor can pass across the Gore membrane of the humidity module, the relative humidity above the sensor is regulated by the *a*_w_ of the LiCl solution (*a*_w_ = RH/100%). In this work, the RH was adjusted in steps of 5% from 15 to 95% RH. The mass of the dry MO film, as well as the amount of water taken up by the lipid film during the water sorption experiments, was calculated according to the Sauerbrey equation. The film thicknesses (*h*) of the dry MO film was roughly estimated from the areal mass of the dry film according to *h* = m_dry_/ρ, assuming an MO density equal to ρ = 1 g/cm^3^.

### 2.3. Sensor Modification

For sensor preparation, interdigitated electrodes (G-IDEAU10) with 10 μm gaps between individual digits (250 digits in total), purchased from Metrohm Autolab B.V. (Utrecht, The Netherlands), were coated with a 10% (*w*/*v*) solution of MO dissolved in ethanol to form films. Before deposition on the IDEs, all samples were vortexed at 2000 rpm. The IDEs were then laminated, leaving a 1 mm diameter punch hole exposed to facilitate drop coating with the lipid solution. This step was to ensure the same area coverage for the IDE. Prior to lamination, the IDEs were cleaned with 99% ethanol and purged with N_2_. Subsequently, 3 μL of MO was drop-cast onto the punch hole, and the IDE was vacuum dried overnight to ensure thorough drying of the thin films. Initial measurements were conducted by varying RH ranges to investigate the response of the MO-coated IDEs to different RH levels.

### 2.4. Wireless Capacitive Measurements

The Adafruit nRF52832 BLE module, a System-on-Chip device, and the Adafruit ADS1115 analog-to-digital converter were sourced from Electrokit (Malmö, Sweden). Additionally, the IDE connector (CACIDE) and interdigitated gold electrodes (G-IDEAU10) were purchased from Metrohm Autolab B.V. (Utrecht, The Netherlands). To conduct wireless capacitance measurements, the Adafruit nRF52832 BLE and Adafruit ADS1115 were housed within a 3D-printed enclosure, with the collected data stored on a smartphone. The Adafruit nRF52832 is a low-energy Bluetooth module that enables wireless data transmission to a smartphone. This board can be powered by a 3.7 V 1400 mAh lithium polymer battery, making it suitable for our specific application (Supplementary Figure S1). The Adafruit ADS1115 offers a 16-bit resolution, which is a significant improvement over the 10-bit resolution of the nRF52832 board, and can measure voltages as low as 76 μV. The mobile phone application used to display capacitance data was developed using MIT App Inventor, while the Arduino code for the nRF52832 was created using the open-source Arduino IDE version 2.1.0 (Supplementary Figure S2).

Initial capacitance measurements were conducted using thin-film-coated IDEs, where the Adafruit nRF52832 BLE module, paired with the ADS1115 16-bit analog-to-digital converter, measured the changes in capacitance. The LiCl solutions were prepared in UHQ water, corresponding to RH values ranging from 12% to 90%. These values correlate with molal concentrations as documented in previous research. RH values were verified using a hygrometer, model 605-H1, from Testo (West Chester, PA, USA), prior to conducting the experiments.

### 2.5. Ex Vivo Wireless Measurements

For SH measurements MO-coated IDEs, a Dermalab^®^ Combo Corneometer, a non-invasive device from Cortex Technology (Aalborg, Denmark), and a BG20 device from Yones Toptech Co. Ltd. (Shenzhen, China) were employed in this study. While MO-coated IDEs assess SH via capacitance measurements, the Dermalab hydration probe operates at a single frequency of 300 kHz to measure skin conductance, which ranges from 0 to 10,000 units, reflecting levels from dry to hydrated skin. The BG20 device assesses SH through a combination of a near-infrared sensor and a thermal conductivity sensor.

Initial capacitance measurements were carried out in a room maintained at 30% RH levels in the room were regulated using a Haze HU-400BCA humidifier from Wilfa (Oslo, Norway). RH levels were monitored with testo 605-H1 hygrometer from Testo (Baden-Württemberg, Germany). ST was recorded before each measurement using a commercial infrared thermometer IP54 from Clas Ohlson (Kunshan, China). IDEs were placed in contact with the skin for ex vivo wireless measurements, and to prevent direct contact with the MO layer, the dialysis membrane was utilized to cover the IDEs. Data collected from the BG20 and the Corneometer were then compared to the capacitance values obtained from the MO-coated IDEs in contact with the skin.

### 2.6. Experimental Replications and Mathematical Modeling

All measurements were performed in triplicate to ensure the reproducibility of the experimental setup. After data analysis, average plot figures were produced using Origin 2017 from OriginLab Corporation (Northampton, MA, USA). In some plots, replicates were depicted to demonstrate data replication with different MO-modified IDEs. To model the mathematical fitting of capacitance as a function of a_w_, a fifth-degree polynomial fit was applied using MATLAB R2022b from MathWorks (Natick, MA, USA).

## 3. Results and Discussion

### 3.1. Wireless Capacitance Measurements

Capacitance measurements began with an Adafruit nRF52832 connected to MO-coated IDEs, capturing shifts in capacitance due to changes in RH, which were recorded on a smartphone via a mobile application (illustrated in Figure 1b). Capacitance (C) is directly proportional to area of cross section (A) and inversely proportional to distance between the gaps of the interdigitated section of the IDE (d), where ε_r_ is the relative permittivity of the thin films coated on IDE and ε_o_ = 8.854 × 10^−12^ F/m is vacuum permittivity, as shown in Equation (2):

(2) $$C=\frac{\varepsilon_{0}\varepsilon_{r}A}{d}$$

This equation underscores the importance of material properties and structural dimensions in determining the capacitance. It is important to note that at ambient condition, *ε,* of liquid water is around 78.4 [63], whereas *ε* of MO is not readily available. However, for a preliminary estimate, it may be assumed that it is similar to other organic molecules, which generally have *ε* ranging from 2 to 10. Therefore, any change in C is predominantly related to the water content in the material occupying the space between the IDEs. The wireless setup, illustrated in Figure 1c, consisting of the Adafruit nRF52832 paired with the ADS1115 analog-to-digital converter, was employed for collecting capacitance data. Initial baseline capacitance measurements were conducted in a custom-designed chamber where the MO-coated IDE (shown in Figure 1d) was purged with nitrogen gas. This chamber was specifically designed to prevent any contact between the MO-coated layer and the solution (as depicted in Figure 1a). Following this, capacitance measurements were taken at various RH levels.

**Figure 2:**
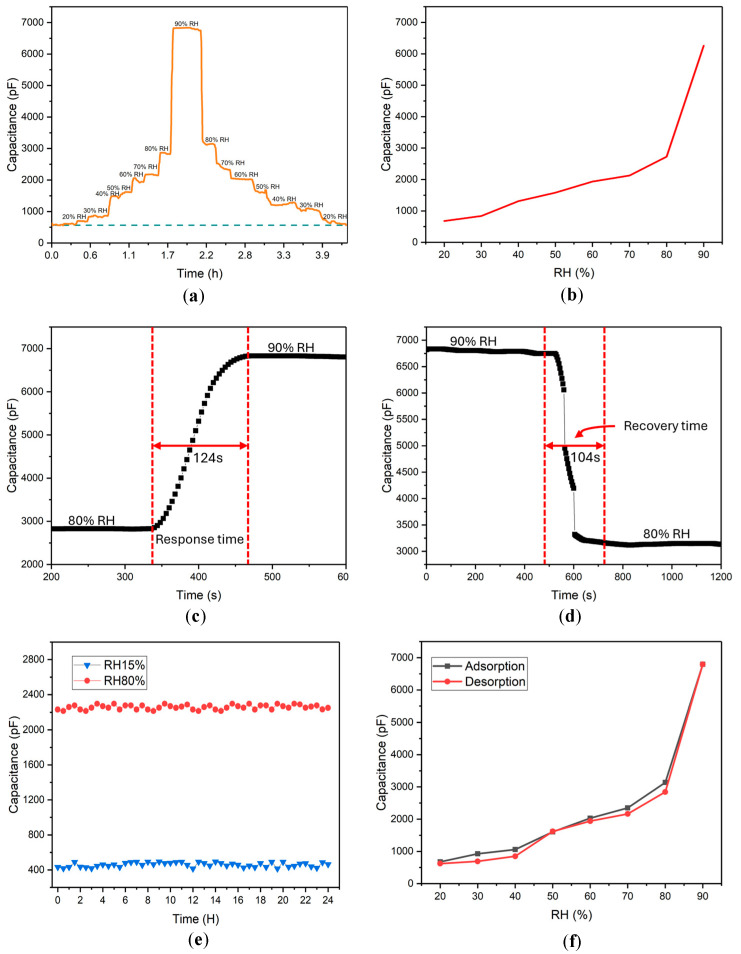
(**a**) Capacitance shift for changing concentration of RH values at RT for MO 10 wt%-coated IDE. (**b**) Typical plot showing capacitance change with increasing RH values. (**c**) Response time and (**d**) recovery time of the MO sensor for RH levels between 80% RH and 90% with the change in capacitance values. (**e**) Typical plot showing capacitance change with time at RH 15% and RH 80%, demonstrating the stability of the sensor platform. (**f**) The hysteresis property of the MO sensor depicted between changing 20% to 90% RH values.

The MO-coated IDEs were initially placed in a nitrogen chamber to establish a baseline capacitance, as shown by the dashed line in Figure 2a. The baseline capacitance in the nitrogen chamber consistently ranged from 400 to 600 pF. This variation is likely due to the variable thickness of the MO layer over the IDEs. The thickness of the MO layer within the 10 μm grooves of the IDEs directly affects the capacitance readings. The MO-coated sensors were prepared by drop-coating a 10% (*w*/*v*) MO solution, and the capacitance was initially measured in the nitrogen chamber. Following this, the change in capacitance was recorded as the RH values were gradually increased from 20% RH to 90% RH and then decreased back to the initial measurement values. Notably, there was no significant change in capacitance at 12% RH, leading to its exclusion from the plot.

At 20% RH, the capacitance stabilized at approximately 620 pF, as shown in Figure 2a. With each subsequent increase in RH, the capacitance also increased. Notably, a significant jump in capacitance was observed from approximately 2800 pF at 80% RH to around 6800 pF at 90% RH. When the RH was subsequently lowered back to 80%, the capacitance decreased to approximately 3127 pF. As RH continued to decrease, the C values returned to levels similar to those observed initially when RH was incrementally increased. This change in capacitance can be attributed to the sorption and desorption of water molecules on the coated MO layer. The response and recovery curve, illustrated in Figure 2a, shows that the C values after decreasing RH levels are similar to those observed during the initial increase in RH. This repeatability was confirmed by performing the response and recovery cycle three times using the same IDEs, which highlights the sensor’s reliability. The response time (61 s) and recovery time (77 s) observed during these measurements were calculated based on the time required to reach 90% of the peak capacitance change, providing insights into the sensor’s dynamics. Furthermore, the fifth-degree polynomial model effectively demonstrates the relationship between water activity (a_w_) and capacitance for the MO-coated IDE, as shown in Supplementary Figure S3. This model captures the non-linear variation in the plot, indicating a positive correlation between a_w_ and capacitance. As water activity increases, capacitance rises at an accelerating rate.

Capacitance measurements with MO-coated IDEs showed an increase in C values as RH levels changed, which can be attributed to the swelling of the MO layer on the IDEs. As the MO layer swells, ε of the film changes, resulting in an alteration of capacitance. When the RH values are subsequently lowered, it leads to the desorption of water molecules from the MO layer, causing a decrease in capacitance. This process results in a shift in capacitance back to values similar to those observed at the beginning of the measurement, ranging from RH 20% to RH 70%, as depicted in Figure 2a. The measurement process concluded with a purge using nitrogen gas, after which the MO-coated IDE returned to its initial C value, demonstrating the sensor’s ability to recover and maintain its baseline performance after exposure to varying humidity levels. Figure 2b shows the increase in capacitance with rising RH values, clearly demonstrating the relationship between RH and capacitance change for the MO-coated IDEs. The sensor’s linear response was calculated, with an R^2^ value of 0.960 within the RH 20% to 80% range, indicating that 96% of the variance in capacitance is explained by the linear model based on RH values (Supplementary Figure S4). The response time and recovery time of the sensor are shown in Figure 2c,d, respectively. The response time for the capacitance to stabilize when the MO-coated IDE was exposed to 90% RH was 124 s, and the recovery time from 90% RH to 80% RH was 104 s. This demonstrates the MO-coated IDE’s faster recovery time in response to decreasing RH.

The stability of the MO-coated IDEs was evaluated over a 24 h period at RH 15% and RH 80%, with the IDEs exposed to LiCl solutions of corresponding molal concentrations. Figure 2e illustrates the stability of the IDE under these distinct RH conditions. Notably, the capacitance remained constant throughout this duration, indicating robust stability under both low and high humidity conditions. MO-coated IDEs are stable as long as the bound vapor molecules on the MO film are removed by vacuum drying. The capacitance returns to the same value when measured in a nitrogen chamber. However, after several runs with the same MO-coated IDE, it was found that the system’s lifetime is 20 ± 2 days. Further testing involved subjecting the MO-coated IDE to increasing RH ranges, followed by a decrease in RH back to the initial measurement values (Figure 2f). These measurements were conducted within the same in-house designed chamber, ensuring consistent environmental conditions. This approach was used to assess the repeatability and reliability of the sensor under cyclic humidity changes, further underscoring the sensor’s durability and performance consistency in fluctuating conditions. Additionally, the hysteresis characteristic of the MO-coated IDEs is shown in Figure 2f. The capacitance trend, which relates to the adsorption and desorption of vapors onto the MO film, leads to an increase in capacitance with rising RH values and a decrease in capacitance with falling RH values.

Capacitance measurements conducted in triplicate for the MO-coated IDEs showed a consistent trend in the evolution of capacitance, as depicted in Supplementary Figure S5. To convert the capacitance values into dimensionless units, the measured C values at various RH levels were divided by the capacitance measured in a nitrogen chamber. This normalization process was implemented to assess the variability among different sensors. Normalized C values for the three IDEs are plotted against RH values ranging from RH 20% to RH 90%, with incremental increases of 10% per step, followed by a decrease of 10% per step returning to the initial value. The capacitance exhibited a relatively slow increase up to RH 80%, followed by a sharp rise at RH 90%. This sharp increase can be attributed to the high water activity within the system, which significantly impacts the capacitance. A directly proportional relationship is evident between the RH and C values of the MO-coated IDEs, indicating a clear correlation between environmental humidity and sensor response.

### 3.2. Water Uptake and Rheological Properties of MO

**Figure 3:**
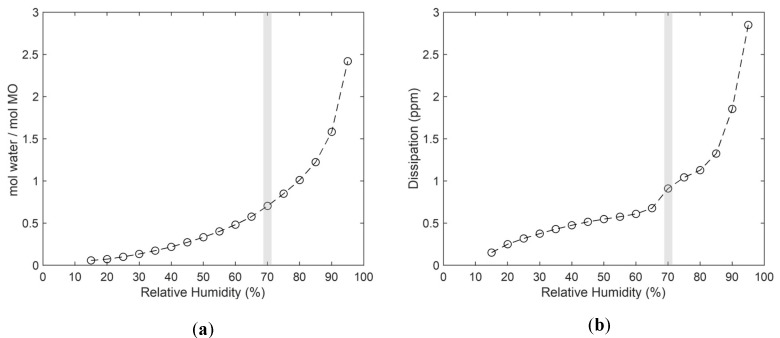
(**a**) Water sorption isotherm and (**b**) dissipation data as a function of RH of MO. Data obtained with QCM-D measurements on an MO film with approximately 100 nm thickness. Data in (**a**) are based on the Sauerbrey equation with overtone 1. Dissipation data are shown for overtone 11. The grey shaded area marks the transition from a solid lamellar phase into a liquid crystalline lamellar phase [60].

Given that the capacitance values are highly dependent on the dielectric properties of the material between the IDEs (as evident from Equation (1)), it was decided to investigate the water uptake of MO at different RH levels using QCM-D. The results from these experiments are summarized in Figure 3a, where the molar ratio is plotted as a function of RH. The graph shows that water uptake ranges from 0 to 2.5 water molecules per MO molecule across the investigated RH range. Notably, the water uptake increases exponentially at higher RH levels, aligning with the pronounced increase in capacitance observed between 80% and 90% RH, as seen in Figure 2. Hypothetically, the capacitance might also depend on the lyotropic phase state of the MO film. Since the dissipation data are highly sensitive for detecting hydration-induced phase transitions, this parameter was also studied as a function of RH. As depicted in Figure 3b, the dissipation data increase with RH. However, the data indicate a distinct change at around 70% RH (highlighted in grey). As previously suggested, this kink is likely related to a phase transition from a solid lamellar phase to a liquid crystalline lamellar phase. Interestingly, this hydration-induced phase transition does not seem to affect the capacitance, which is apparently more sensitive to the amount of water rather than the physical state of the MO molecules.

### 3.3. Variable Temperature–Capacitance Measurements

**Figure 4:**
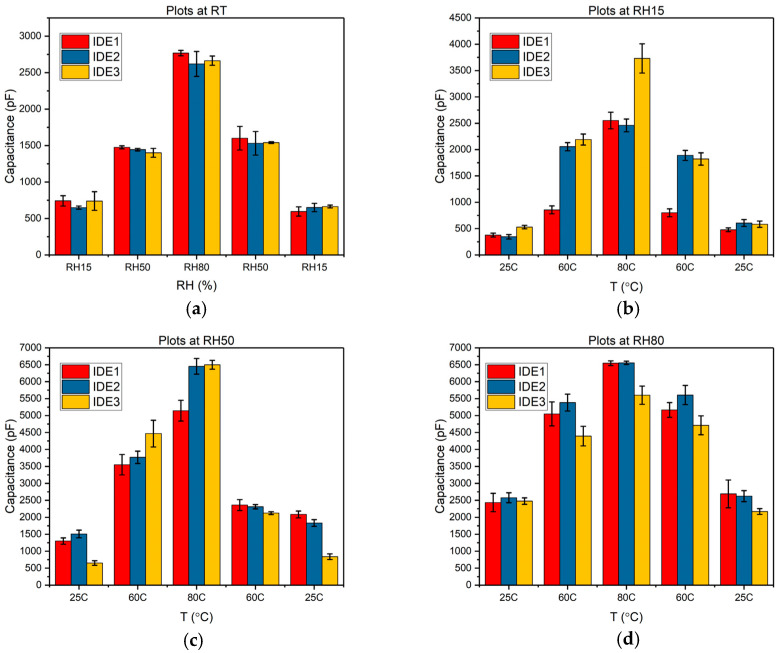
(**a**) Wireless capacitance measurement with MO (10 wt%)-coated IDE, depicting the change in capacitance at RT with varying RH only. (**b**) Wireless capacitance measurements at a fixed RH of 15%, displaying the change in capacitance with temperature variations only. Subsequently, capacitance measurements were conducted at a fixed RH of 50% (**c**) with varying temperature values. (**d**) Capacitance measurement with the MO-coated sensor at an RH of 80%, with selective temperature changes.

Initial capacitance measurements with MO-coated IDEs were carried out at room temperature (RT), as illustrated in Figure 4a. The IDEs were initially exposed to selective RH values, with baseline capacitance measurements conducted in a nitrogen chamber before the IDEs were subjected to increasing RH levels. There was a slight increase in capacitance at 15% RH, followed by progressively larger increases up to the 80% RH range. As the RH values subsequently decreased, the C values also diminished, returning to levels comparable to those observed during the initial rise in RH. The results of these measurements, conducted in triplicate at room temperature with three different MO-coated IDEs, are depicted in Figure 4a, showing consistent repeatability and stability of the sensor response under varying humidity conditions.

At a fixed RH of 15%, further measurements were conducted with variations in temperature alone, ranging from 25 °C to 80 °C, as depicted in Figure 4b. There was a noticeable initial increase in capacitance, followed by a further increase at 80 °C. Notably, when the temperature was subsequently decreased, the sensor capacitance almost returned to its initial values. This behavior can be attributed to the thermotropic phases of MO, which cause phase transitions in the MO structure, affecting the capacitance. Similarly, in Figure 4c, at a constant RH of 50%, the temperature alone was varied while the MO-coated IDE was exposed to a fixed molal concentration of LiCl solution. Here, the capacitance trended upwards until reaching 80 °C but then showed a lower value compared to the initial range of around 4500 pF at 60 °C.

This decrease is likely due to a temperature-induced phase transition in the MO film on the IDEs. For the RH 80% scenario with temperature-varying capacitance measurement (Figure 4d), an increase in capacitance was observed up to 80 °C. However, a decreasing trend was noted when the temperature values were subsequently reduced. This pattern demonstrates the reversibility of the sensor platform, indicating its potential for reliable and repeated use in varying environmental conditions. This reversibility is a crucial attribute for sensors operating under diverse temperature and humidity conditions, enhancing their utility across different applications.

### 3.4. Wireless Capacitive Skin Hydration Measurements

**Figure 5:**
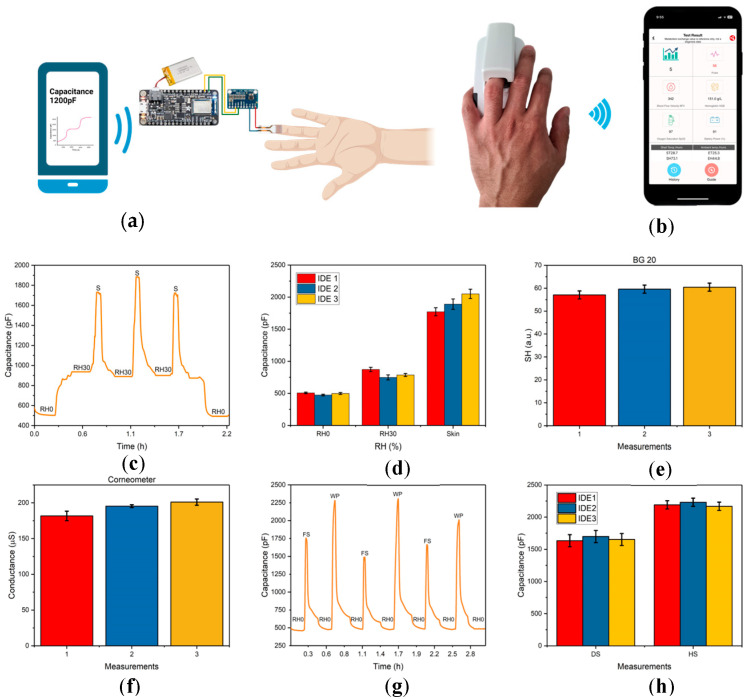
Capacitance measurements using MO-coated IDE-based device compared to SH values and conductance values obtained using BG20 device and Dermalab^®^ Combo Corneometer. (**a**) Schematic visualization for Bluetooth-based wireless capacitance measurements, depicting proof of concept where MO-coated IDE functions as humidity sensor and the data can be collected on the phone (created with BioRender.com). (**b**) Visualization of wireless BG20 device. (**c**) Typical plot illustrating response/recovery cycle of capacitance with changing RH values transitioning from a nitrogen chamber to RH 30%, followed by fingertip contact (S). (**d**) Measurements were performed in triplicate with three MO-coated IDEs. SH measurements were performed in triplicate with control studies using (**e**) wireless BG20 and (**f**) wired Corneometer hydration probe. (**g**) The change in capacitance with MO-coated IDE in contact with forearm skin (FS) and after placing wet patch (WP) on the skin. (**h**) Measurements depict the change in capacitance with MO-coated IDEs in contact with dry skin (DS) and hydrated skin (HS) after the application of a wet patch over the left forearm.

Wireless measurements were carried out using the nRF52832 microcontroller, as shown in Figure 5a, alongside the commercially available BG20 wireless device, depicted in Figure 5b. Accuracy of the environmental conditions was ensured through monitoring with testo 605-H1 hygrometer. The process began with initial capacitance measurements conducted in a nitrogen chamber, followed by further measurements at a consistently maintained 30% RH, using the humidifier in the room. The typical response and recovery cycle of the MO-coated IDE is shown in Figure 5c, illustrating the change in capacitance when the IDE is in contact with the skin (S). After removing the IDE from the skin and leaving it at RH 30%, the C value returned to a lower level, similar to that observed before skin contact. This demonstrates the sensor’s ability to recover and stabilize after use.

The changes in capacitance depicted in Figure 5c can indeed be attributed to variations in SH. When the sensor comes into direct contact with the skin, it alters ε of the MO layer, leading to an increase in capacitance. This behavior is clearly illustrated in Figure 5c, where the capacitance peaks at a value similar to the initial measurement when the sensor is in contact with the skin. This demonstrates the reversibility of the MO-coated sensor’s response, as the capacitance increases upon skin contact and then decreases to a level comparable to that observed before the measurement at 30% relative humidity (RH), as monitored by the hygrometer. Figure 5d displays measurements performed in triplicate with three different MO-coated IDEs. Each set shows the average of three measurements taken with each IDE, labeled as IDE 1, 2, and 3. This setup is designed to illustrate the variability between measurements, highlighting the repeatability and reliability of the sensor across multiple tests and conditions. Such detailed analysis helps underscore the sensor’s consistent performance and its potential utility in various practical applications where monitoring SH is critical.

To effectively compare the capacitance values with those from commercially available gadgets, the same part of the fingertip was examined using both the wireless BG20 device and the wire-based Dermalab^®^ Corneometer. The experimental results from the BG20 in Figure 5e represent measurements in triplicate of skin hydration, which were conducted alongside measurements from the Dermalab^®^ Combo, as shown in Figure 5f. While the MO-coated IDEs measure changes in capacitance related to SH, the BG20 and Corneometer assess changes in the electrical properties of the skin. Specifically, the BG20 measures thermal conductivity and converts these readings into arbitrary units (a.u.), whereas the Corneometer measures skin conductance in microsiemens (μS), which aids in evaluating skin dryness or hydration. These differing methodologies highlight the distinct approaches each device uses to measure SH, providing valuable insights into their respective accuracies and applications. The grouped bar plots for skin measurements (Figure 5d) show an increase in capacitance values for three different IDEs. This increase correlates with the skin hydration values measured using the BG20 (Figure 5e) and the Corneometer (Figure 5f), suggesting a relationship between the capacitance measured by the MO-coated IDEs and the SH measurements from both the BG20 and Corneometer. According to the manufacturer of the Dermalab^®^ Combo, the measured skin hydration value of 192 ± 9 μS from the average of three bar plots in Figure 5f corresponds to moderately dry skin. Conversely, the BG20 reports a value of 59 ± 1 a.u., indicating moderately hydrated skin (Figure 5e). Previous studies have demonstrated relatively better accuracy and precision with the Corneometer hydration probe. Given that exposure of the skin to 30% RH typically leads to dryness, the measurements from the Corneometer are considered more precise and accurate for this specific condition. This comparative analysis emphasizes the utility of the MO-coated IDE in providing reliable measurements that are consistent with established methods of assessing SH, further validating its potential use in various applications where real-time, precise monitoring of skin conditions is essential.

Capacitance measurements using MO-coated IDEs were systematically performed to assess the impact of hydration changes on the skin, with the results detailed in Figure 5g. The initial measurements were conducted in a nitrogen chamber, marked as RH0, indicating a baseline environment with no additional humidity. Subsequent measurements were taken in direct contact with the skin on the left forearm (FS). Before applying the IDE to the FS, ST and conductance were recorded. The IR thermometer showed an ST of 31 ± 0.5 °C, and the Corneometer measured skin conductance at 135 ± 2.5 μS, suggesting a baseline level of skin dryness. Following the application of a wet patch (WP) on the FS, the ST decreased to 27 ± 0.45 °C, and the skin conductance increased significantly to 458 ± 9.45 μS, indicating increased hydration. The capacitance of the FS was initially measured at 1700 ± 62 pF prior to applying the WP. After the WP was applied to the left FS for 2 min, the capacitance rose to 2302 ± 182 pF. This substantial increase in capacitance post-application of the WP clearly demonstrates the sensor’s sensitivity to changes in skin hydration. This is further illustrated in Figure 5h, which shows triplicate capacitance measurements before and after applying the WP for 2 min. A clear change in capacitance is evident, viz., the *C* value measured before application of WP on FS was 1633 ± 93 pF, which increased to 2191 ± 64.2 pF after applying the WP. Moreover, the correlation between the capacitance values and the skin conductance measurements suggests that the skin was relatively dry before the WP application and became significantly more hydrated afterward. This relationship underscores the efficacy of using capacitance measurements to gauge SH levels, validated by corresponding changes in conductance. These findings highlight the potential of MO-coated IDEs in real-time, wireless, non-invasive monitoring of SH, beneficial for applications in healthcare, cosmetic testing, and sports science.

**Figure 6:**
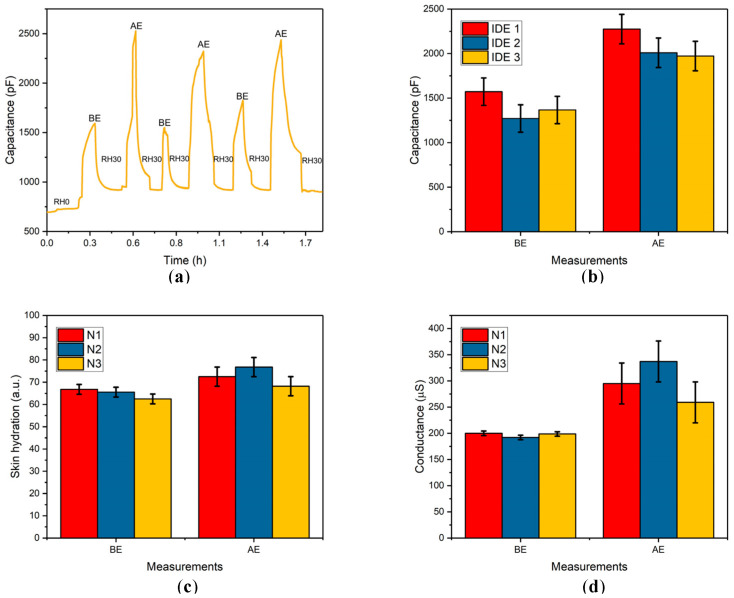
Data illustrating changes in different measured parameters for SH before exercise (BE) and after exercise (AE) by two commercially available devices and MO-coated IDEs. (**a**) The response/recovery cycle of the MO-coated IDE observed BE and AE, when the MO-coated IDEs were in contact with the fingertip. (**b**) Measurements in triplicate of capacitance with different IDEs in contact with the fingertip BE and AE. (**c**) Measurements in triplicate of SH BE and AE using BG20 device with the fingertip. (**d**) Measurements in triplicate of skin conductance by Dermalab^®^ Combo Corneometer BE and AE over the left forearm.

Capacitance measurements were conducted to assess the effect of rigorous exercise on SH. As depicted in Figure 6a, capacitance measurement was performed with contact to the left index fingertip. The capacitance measurement BE yielded 1672 ± 127 pF, whereas AE, it increased to 2440 ± 87 pF. Additionally, the capacitance was 922 ± 15 pF at room temperature (RT) after IDE recovery. This plot illustrates the general behavior of the sensor. The room temperature was 25 °C, with RH30% continuously monitored throughout the experiment by a hygrometer. ST was also measured by an IR thermometer, indicating a temperature difference of approximately 3 °C after exercise. The same capacitance measurements were performed to assess the repeatability of the response-to-recovery cycle (Supplementary Figure S6). As illustrated in Figure 6b, an increase in capacitance was observed after exercise, attributed to a change in ε of the coated MO layer. This was further confirmed by repeated capacitance measurements with MO-coated IDEs, which also showed similar trends in capacitance values.

The BG20 and the Dermalab^®^ Combo devices were used to measure SH before exercise (BE) and after exercise (AE) to evaluate the correlation between changes in capacitance and actual SH values. The data are presented in Figure 6c for the BG20 and Figure 6d for the Corneometer. BE, the skin conductance measured by the Corneometer was 200 ± 4 μS, which increased to 295 ± 39 μS AE, indicating a significant increase in SH. The BG20 device also showed an increase, with SH measurements rising from 67 ± 2 a.u. BE to 72 ± 4 a.u. AE. These results highlight a measurable increase in SH following physical activity, which is expected due to increased sweating and the activation of various physiological processes that impact skin moisture levels. Additionally, the capacitance measurements made with the MO-coated IDEs also reflected changes corresponding to the exercise-induced hydration increase. As shown in Figure 6b,d, the increase in capacitance is directly proportional to the increase in skin conductance measured by the Corneometer. This correlation suggests that the capacitance changes observed with the MO-coated IDEs accurately reflect changes in SH, validating the use of these sensors for monitoring SH. This direct relationship between capacitance and skin conductance underscores the effectiveness of using MO-coated IDEs for real-time, non-invasive monitoring of SH changes associated with physical activity. The skin conductance measurements in triplicate presented in Figure 6d can be compared with the capacitance measurements in triplicate shown in Figure 6b, both of which exhibit similar trends BE and AE. Since the Dermalab^®^ Combo is a commercially accepted device for SH measurements, this comparison suggests that the MO-modified IDEs demonstrate repeatability and reliability as a sensing platform. Such sensors provide valuable data that can be used in various fields, including sports science, healthcare, and wellness, to monitor physiological responses to different conditions and activities.

In summary, this study introduces a novel humidity sensor platform, specifically designed to assess SH. The stability of the sensor was validated through capacitance measurements taken at 15% and 80% RH over a 24 h period. Additionally, the response–recovery cycle provided valuable insights into the sensor’s response time (61 s) and recovery rate (77 s) during skin measurements, highlighting its efficient dynamics. The sensor demonstrated robust reusability through three consecutive measurements, affirming its potential for repeated use in practical scenarios. Correlation analysis indicated a direct relationship between the capacitance values measured by the MO-coated IDEs and those obtained from commercially available devices, particularly with the Corneometer’s skin conductance values. Notably, SH measured at a maintained 30% RH indicated moderately dry skin according to the Corneometer, although findings from the BG20 were less definitive, suggesting the need for careful interpretation of the data from different devices. Furthermore, post-exercise SH measurements conducted with the Corneometer, alongside capacitance changes detected by the MO-coated sensor, exhibited a clear correlation between changes in capacitance and skin conductance. This demonstrates the sensor’s capability to accurately reflect physiological changes in real-time. Capacitance measurements performed on the forearm skin (FS), both before and after the application of a wet patch (WP), conclusively showed that SH significantly influences capacitance levels. These findings validate the effectiveness of the MO-based sensor in providing accurate, real-time insights into SH under various conditions, confirming its potential utility in diverse applications such as the healthcare, sports, and cosmetic industries.

## 4. Conclusions

This work introduces a novel type of capacitive humidity sensor that is capable of wireless measurements of both RH and SH, thanks to an amphiphilic film, specifically MO, a promising material for humidity sensing. First, the properties of MO were characterized using QCM-D to establish the correlation between RH and phase transitions. The experimental results indicated that the capacitance of the system was primarily influenced by the amount of water within the MO system, while the lyotropic or physical state of the amphiphilic film played a secondary role. Second, tests across different RH values (from 0% to 90% RH) demonstrated that the sensor effectively responded to changes above 20%, also showcasing its reversible nature. The sensitivity of the developed sensor was determined to be 7 pF/%RH, and good linearity with an R^2^ value of 0.960 within the RH 20% to 80% range was calculated. The dynamic response and recovery times were measured to be 124 and 104 s, respectively. The study highlights the sensor’s sensitivity to fluctuations in both temperature and a range of RH values, demonstrating its quick response to changes in humidity. This confirms the stability and reliability of the sensor design under various environmental conditions. Third, the performance of the developed sensor was compared to two commercially available devices—one wireless and one wire-based—confirming the functionality of the MO-based IDE sensor. Specifically, triplicate measurements of SH levels of the volunteers showed similar trends, and SH values of the fingertips measured by the MO-based IDE sensor and BG20 device are in good agreement with each other. The findings from the tests before and after exercises validated the effectiveness of the MO-based sensor in providing accurate, real-time insights into SH under various conditions. Fourth, the developed sensor platform’s reusability was confirmed to exceed 18 days. This further demonstrates the robustness of the sensor and its suitability for repeated applications for both SH and RH measurements. Last but not least, a key advantage of this sensor is its capacity for wireless data transmission and collection, enabling real-time measurements in diverse environments. This makes it particularly useful for applications in healthcare, sports science, and personal care, where continuous monitoring of physiological parameters is crucial.

## Data Availability

The data that support the findings of this study are available from the corresponding authors upon reasonable request.

## Supplementary Materials

The following supporting information can be downloaded at: https://www.mdpi.com/article/10.3390/s24144449/s1, Figure S1: The Adafruit nRF52832 (left) connected with ADS1115 analog-to-digital converter. Figure S2: The Arduino code for programming the Adafruit nRF52832. Figure S3: Plot illustrating the relationship between capacitance and *a*_w_, accompanied by the model-fitting equation to provide a detailed explanation. Figure S4: Plot illustrating the linear response of the MO-based sensor to varying RH values. Figure S5: Normalized capacitance data for the IDEs for different RH values. Figure S6: Wireless capacitance measurements with the MO-coated IDE BE and AE showing change in capacitance.
