# Supplementary material for: Monoolein-Based Wireless Capacitive Sensor for Probing Skin Hydration
Source: Sensors (Basel). 2024 Jul 10;24(14):4449. doi: 10.3390/s24144449 (PMC11280606; doi:10.3390/s24144449)
Supplement: Supplementary file 1 [file sensors-24-04449-s001.zip › sensors-3050076-supplementary.pdf]

Monoolein-Based Wireless Capacitive Sensor for Probing Skin Hydration

Vivek Chaturvedi [1,2], Magnus Falk [1,2,*], Sebastian Björklund [1,2], Juan F. Gonzalez-Martinez [1,3] and Sergey Shleev [1,2,*]

[1] Department of Biomedical Science, Faculty of Health and Society, Malmö University, 20506 Malmö, Sweden; vivek.chaturvedi@mau.se (V.C.); sebastian.bjorklund@mau.se (S.B.); juan.fransisco.gonzales@mau.se (J.F.G.-M.)
[2] Biofilms Research Center for Biointerfaces, Malmö University, 20506 Malmö, Sweden
[3] Department of Applied Physics and Naval Technology, Polytechnical University of Cartagena, 30202 Cartagena, Spain
* Correspondence: magnus.falk@mau.se (M.F.); sergey.shleev@mau.se (S.S.)

Supplementary Material

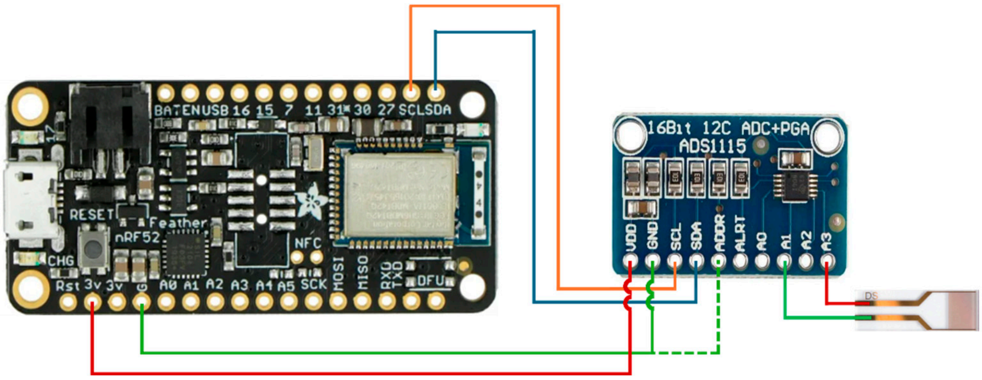

Supplementary Figure S1. The Adafruit nRF52832 (left) connected with ADS1115 analog to digital converter. The ADS 1115 (right) connects monoolein coated inter digitated electrode.

Code for Adafruit nRF52832

```
#include <bluefruit.h>
#include <avr/dtostrf.h>
#include <Wire.h>
#include <Adafruit_ADS1X15.h>


// OTA DFU service
BLEDfu bledfu;

// Uart over BLE service
BLEUart bleuart;

// Function prototypes for packetparser.cpp
uint8_t readPacket (BLEUart *ble_uart, uint16_t timeout);
float   parsefloat (uint8_t *buffer);
void    printHex   (const uint8_t * data, const uint32_t numBytes);

// Packet buffer
extern uint8_t packetbuffer[];

const int OUT_PIN = A2;
const int IN_PIN = A0;
const float IN_STRAY_CAP_TO_GND = 930.0;
const float IN_CAP_TO_GND  = IN_STRAY_CAP_TO_GND;
const float R_PULLUP = 136000.0;
const int MAX_ADC_VALUE = 65535;


float term1 = 6600.0;

float term2 = 22562.91079812207;
float term3 = 119852.02939698334;
```

```
float term4 = 1.1829224940165663e6;
float term5 = 3.8847703348326143e6;
float term6 = 1.080521450758494e8;
float term7 = 4.8557090453618914e8;
float term8 = 4.773925189760303e9;
float term9 = 1.874649021586866e10;
float term10 = -2.857744934788257e11;
float term11 = -6.813492087090683e12;
float term12 = -4.358005855987904e13;
float term13 = -7.0245813489014e14;




Adafruit_ADS1115 ads1115;

const int numReadings = 30;    // Number of readings to take

float readings[numReadings];     // Array to store the readings
int ind = 0;
float total = 0;                 // Sum of all readings

void setup() {
  Serial.begin(115200);

  Bluefruit.begin();
  Bluefruit.setTxPower(4);    // Check bluefruit.h for supported values
  Bluefruit.setName("Bluefruit52");

  // To be consistent OTA DFU should be added first if it exists
  bledfu.begin();

  // Configure and start the BLE Uart service
  bleuart.begin();

  // Set up and start advertising
  startAdv();

  ads1115.setGain(GAIN_ONE);
  ads1115.begin();
//  analogReadResolution(14);
  pinMode(OUT_PIN, OUTPUT);
  pinMode(IN_PIN, OUTPUT);

  for (int i = 0; i < numReadings; i++) {
    readings[i] = 0;
  }
}
```

```
void startAdv(void)
{
  // Advertising packet
  Bluefruit.Advertising.addFlags(BLE_GAP_ADV_FLAGS_LE_ONLY_GENERAL_DISC_MODE);
  Bluefruit.Advertising.addTxPower();

  // Include the BLE UART (AKA 'NUS') 128-bit UUID
  Bluefruit.Advertising.addService(bleuart);

  // Secondary Scan Response packet (optional)
  // Since there is no room for 'Name' in Advertising packet
  Bluefruit.ScanResponse.addName();

  /* Start Advertising
   * - Enable auto advertising if disconnected
   * - Interval:  fast mode = 20 ms, slow mode = 152.5 ms
   * - Timeout for fast mode is 30 seconds
   * - Start(timeout) with timeout = 0 will advertise forever (until connected)
   *
   * For recommended advertising interval
   * https://developer.apple.com/library/content/qa/qa1931/_index.html
   */
  Bluefruit.Advertising.restartOnDisconnect(true);
  Bluefruit.Advertising.setInterval(32, 244);    // in unit of 0.625 ms
  Bluefruit.Advertising.setFastTimeout(30);      // number of seconds in fast mode
  Bluefruit.Advertising.start(0);                // 0 = Don't stop advertising after n seconds
}

void loop() {

  int16_t adc0;
  pinMode(IN_PIN, INPUT);
  analogWrite(OUT_PIN, 255);
  //int val = analogRead(IN_PIN);
  int16_t val = ads1115.readADC_SingleEnded(0);
  analogWrite(OUT_PIN, 0);


  if (val < 24000) {
    pinMode(IN_PIN, OUTPUT);

    float valf = (float)val/(float)MAX_ADC_VALUE;

  // float capacitance =(float)-493.766+0.0024589*exp(0.420763 +38.7611*valf)-1137.22*sqrt(-0.0709545+valf)+7583.54*valf;
  float capacitance =  -352310.4353945729 +
        2.710359649734561e7 * valf -
        9.159161885614538e8 * pow(valf, 2) +
        1.7893611760694912e10 * pow(valf, 3) -
        2.2349472075353256e11 * pow(valf, 4) +
```

```
      1.8564099890381064e12 * pow(valf, 5) -
      1.0233418047023395e13 * pow(valf, 6) +
      3.5090199022765117e13 * pow(valf, 7) -
      5.416939045860601e13 * pow(valf, 8) -
      9.853537521250548e13 * pow(valf, 9) +
      7.287411627998412e14 * pow(valf, 10) -
      1.714517188261953e15 * pow(valf, 11) +
      1.9862779975892225e15 * pow(valf, 12) -
      9.489784707132432e14 * pow(valf, 13);
    //float capacitance = -(float)t/(R_PULLUP*log(1.0-(float)val/(float)MAX_ADC_VALUE));
  readings[ind] = capacitance;
  total += readings[ind];
  // Move to the next index in the array
  ind = (ind + 1);
  if ((ind-(numReadings-1)) == 0) {

    // Calculate the mean
    float mean = (float)total / numReadings;
    // Calculate the sum of the squared differences from the mean
    float sumSquaredDiff = 0.0;
    for (int i = 0; i < numReadings-1; i++) {
      float diff = readings[i] - mean;
      sumSquaredDiff += diff * diff;
    }
    // Calculate the standard deviation
    float standardDeviation = sqrt(sumSquaredDiff / numReadings);

    //      Serial.print(F("Capacitance Value = "));
    //float capacitance = 455.45924592806153-433.2314905571894*exp(-0.0026865737775692766*mean);
    //float capacitance = 2.53600603823788*exp(-0.47035522404354063+0.016253492241958163*mean);
    //float capacitante = mean;
    Serial.print(capacitance,0);
    Serial.print(",");
    Serial.println(standardDeviation,0);
    bleuart.print(String(capacitance));
    //      Serial.print(F(" pF ("));
    //      Serial.print(val);
    //      Serial.println(F(") "));
    ind = 0;
    for (int i = 0; i < numReadings; i++) {
      readings[i] = 0;
    }
    total = 0.0;
    sumSquaredDiff =0.0;
  }
}

else {
  pinMode(IN_PIN, OUTPUT);
```

```
    delay(1);
    pinMode(OUT_PIN, INPUT_PULLUP);
    unsigned long u1 = micros();
    unsigned long t;
    int digVal;

    do {
      digVal = ads1115.readADC_SingleEnded(0);
      unsigned long u2 = micros();
      t = u2 > u1 ? u2 - u1 : u1 - u2;
    } while ((digVal < 20000) && (t < 400000L));

    pinMode(OUT_PIN, INPUT);
    val = ads1115.readADC_SingleEnded(0);
    analogWrite(IN_PIN, 255);
    int dischargeTime = (int)(t / 1000L) * 5;
    delay(dischargeTime);
    pinMode(OUT_PIN, OUTPUT);
    analogWrite(OUT_PIN, 0);
    analogWrite(IN_PIN, 0);

    float capacitance = -(float)t / R_PULLUP / log(1.0 - (float)val / (float)MAX_ADC_VALUE);

    Serial.print(F("Capacitance Value = "));
    if (capacitance > 1000.0) {
      Serial.print(capacitance / 1000.0, 2);
      Serial.print(F(" uF"));
    }
    else {
      Serial.print(capacitance, 2);
      Serial.print(F(" nF"));
    }

    Serial.print(F(" ("));
    Serial.print(digVal == 1 ? F("Normal") : F("HighVal"));
    Serial.print(F(", t= "));
    Serial.print(t);
    Serial.print(F(" us, ADC= "));
    Serial.print(val);
    Serial.println(F(")"));
    bleuart.print(String(capacitance));
  }
  while (micros() % 100 != 0);

  //delayMicroseconds(100);
}
```

Supplementary Figure S2. Arduino code for capacitance measurements were uploaded in Adafruit nRF52832 using version Arduino IDE 2.1.0.

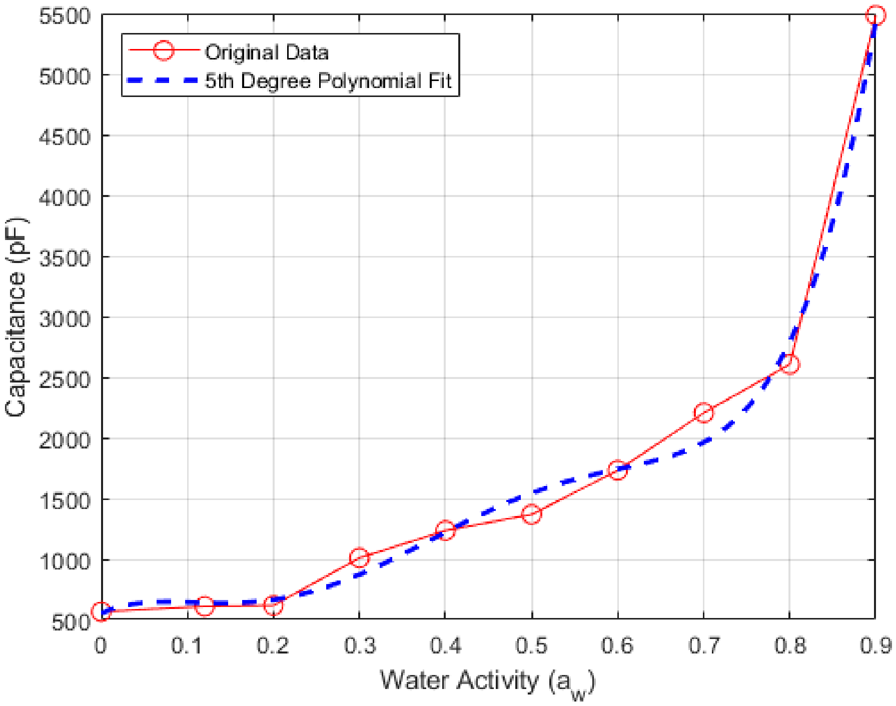

Supplementary Figure S3. Capacitance as a function of a_w demonstrated by a fifth-degree polynomial fit.

The fifth-degree polynomial fit model is well-suited for capturing the dataset's behavior, offering a nuanced and accurate representation of the relationship between a_w and capacitance (Supplementary Figure S3.). Analyzing the coefficients in detail helps uncover the underlying patterns in the data, providing valuable insights into how capacitance changes with varying levels of water activity. The plot clearly shows a positive correlation between a_w and capacitance. The polynomial equation utilized to model the relationship between capacitance (C) and water activity (a_w), along with the associated coefficients, is provided in Supplementary Equation S1 below.

$$C(a_w) = p_1.a_w^5 + p_2.a_w^4 + p_3.a_w^3 + p_4.a_w^2 + p_5.a_w + p_6 \quad \text{(S1)}$$

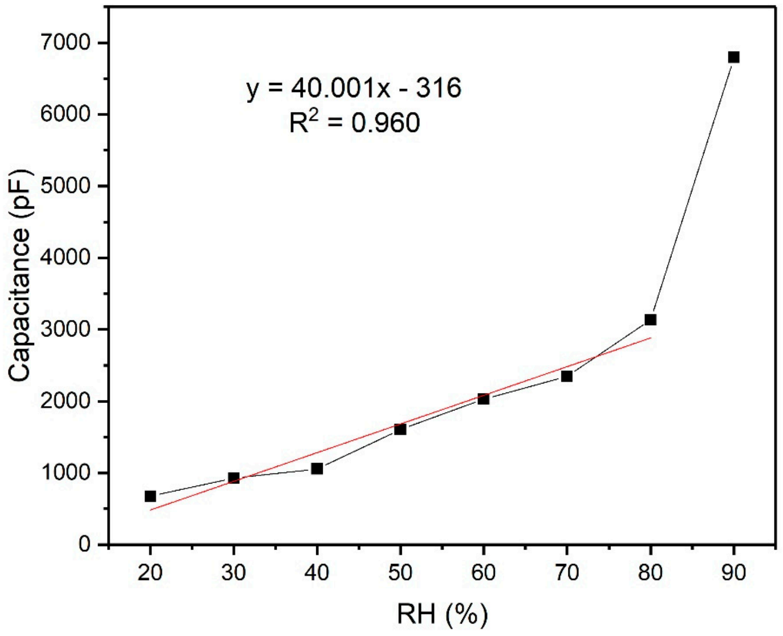

Supplementary Figure S4. Plot illustrating the linear response of the MO-based sensor to varying RH values. The R^2 value indicates that 96% of the variance in capacitance is explained by the linear model based on relative humidity.

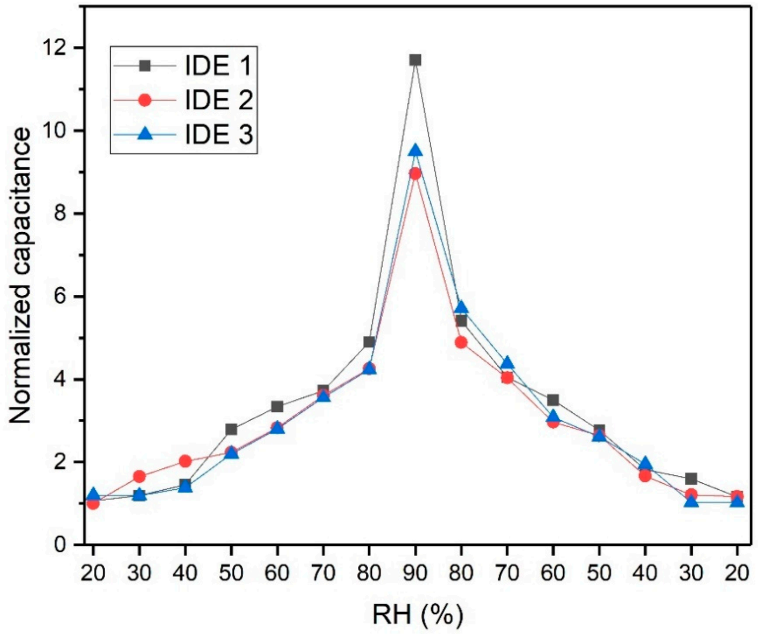

Supplementary Figure S5. Normalized capacitance data for the IDEs for different RH values.

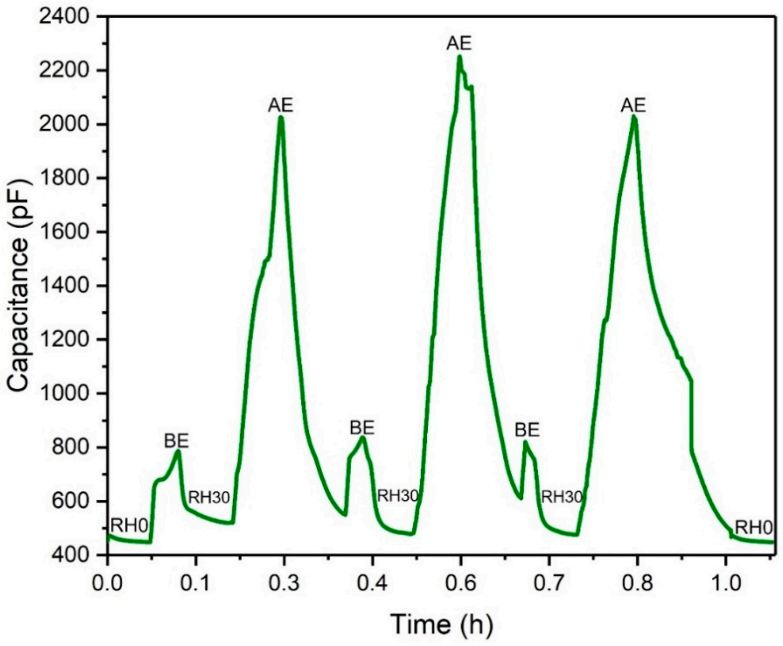

Supplementary Figure S6. Wireless capacitance measurements with the MO-coated IDE before exercise (BE) and after exercise (AE) showing change in capacitance.
